# Fumigant, contact, and repellent activities of essential oils against the darkling beetle, *Alphitobius diaperinus*

**DOI:** 10.1093/jis/14.1.75

**Published:** 2014-01-01

**Authors:** Xuegui Wang, Qian Li, Litao Shen, Jizhi Yang, Huabao Cheng, Surong Jiang, Chunxian Jiang, Haijian Wang

**Affiliations:** 1 Sichuan Agricultural University, Biorational Pesticide Research Lab, 611130, Chengdu, China; 2 Sichuan Agricultural University, College of Resources and Environment, 611130, Chengdu, China

**Keywords:** AChE activity, fumigant toxicity, repellent toxicity

## Abstract

The fumigant, contact, and repellent activities of four essential oils extracted from *Citrus limonum* (Sapindales: Rutaceae), *Litsea cubeba* (Laurales: Lauraceae), *Cinnamomum cassia* , and *Allium sativum* L. (Asparagales: Alliaceae) against 6th instars and adults of the darkling beetle, *Alphitobius diaperinus* (Panzer) (Coleoptera: Tenebrionidae), one of the main pests of materials and products of *Juncus effuses* L. (Poales: Juncaceae) during the storage period, were assayed, and chemical ingredients were analyzed with gas chromatography-mass spectrometry in this study. While the major ingredients found in *C. limonum* and *C. cassia* were limonene and (E)- cinnamaldehyde, the main constituents of *L. cubea* were D-limonene, (E)-3,7-dimethyl-,2,6-octadienal, (Z)-3,7-dimethyl,2 ,6-octadienal, and diallyl disulphide (18.20%), while the main constituents of and *A. sativum* were di-2-propenyl trisulfide and di-2-propenyl tetrasulfide. The fumigation activities of *A. sativum* and *C. limonum* on *A. diaperinus* adults were better than those of the other two essential oilss. The toxicities of *A. sativum* and *C. limonum* were almost equitoxic at 96 hr after treatment. Essential oils from *Allium sativum* and *L. cubeba* also showed good contact activities from 24 hr to 48 hr, and toxicities were almost equitoxic 48 hr posttreatment. The repellent activities of *A. sativum* and *L. cubeba* oils on 6th instars were also observed, showing repellence indexes of 90.4% and 88.9% at 12 hr after treatment, respectively. The effects of *A. sativum* on AChE activity of 6th instars of *A. diaperinus* were strongest compared to the other essential oils, followed by *C. limonum* , *L. cubeba,* and *C. cassia* . These results suggest that the essential oils of *C. limonum* and *A. sativum* could serve as effective control agents of *A. diaperinus.*

## Introduction

At present, *Juncus effuses* L. (Poales: Juncaceae) is being widely cultivated in southwest China and is used as an important material for summer sleeping mats; it is exported to Japan, Korea, and some other southeast Asian countries, generating about $50 million per year in revenue ( Li et al. 2009 ). *Alphitobius diaperinus* (Panzer) (Coleoptera: Tenebrionidae) is one of the most destructive pests of *J. effuses* in storage, and control treatments heavily depend on the use of synthetic insecticides and fumigants. However, excessive chemical applications have serious health hazards for human applicators, customers’ skins, the environment, etc. ( Wang et al. 2011 ). These problems required the developmentment of selective, environmentally-acceptable insect control alternatives ( Copping and Menn 2000 ). Plant-derived natural chemicals have been known as secondary metabolites and possible alternatives to synthetic chemical insecticides and displayed strong biological activities ( Cosimi et al. 2009 ; Nesci et al. 2011 ). In recent years, the search for new plant oils with insecticidal activity as possible alternatives to synthetic chemical insecticides has become a hotspot in pesticide research ( Stefanazzi et al. 2011 ). Essential oils (EO) are volatile, small molecule, complex secondary metabolites that have been widely researched for their fumigant, contact, and repellant activities and have been developed as stored-product pest repellents or antifeedants ( Jin et al. 2010 ).

The aim of this study was to determine the main components and examine the insecticidal activities of EOs extracted from four aromatic plants, *Citrus limonum* (Sapindales: Rutaceae), *Litsea cubeba* (Laurales: Lauraceae), *Cinnamomum cassia* , and *Allium sativum* L. (Asparagales: Alliaceae), against *A. diaperi-nus* and the effects on AChE activity of *A. diaperinus* . The results will give an insight into the potential of the tested EOs as effective alternatives to synthetic insecticides against *A. diaperinus* occurring storage.

## Materials and Methods

### Insect cultures

Larvae and adults of *A. diaperinus* were reared in laboratory and maintained in darkness in incubators at 27 ± 2°C and 70 ± 5% RH. They were fed with a mix of wheat bran, maize powder, and peanut cake at an 8:1:1 weight proportion and 15% water. Sixth instars and adults two days post-eclosion were used in all experiments.

### Essential oil extraction and gas chromatography-mass spectrometry

Leaves of *C. limonum, L. cubeba* , *C. cassia* , and *A. sativum* were collected during the autumn from Wenjiang District, Chengdu, China, located at 30°41ʹ′48.23ʺ″ N, 103°49ʹ′57.46ʺ″ E. The EOs were extracted with hydrodistillation using a modified Clevenger apparatus about 3–4 hr, dried over anhydrous sodium sulfate, and refrigerated at 4°C ( Mahnaz et al. 2012 ).

The oils were analyzed in authors’ laboratory by gas chromatography-mass spectrometry (GC-MS) with an Agilent 6890 chromatograph (( www.agilent.com ) connected to a 5973N mass spectrometer equipped with a capillary column (HP-5, 30 m × 0.25 mm, 0.25 µm). The GC oven temperature was held at 50°C for 2 min, programmed at 5°C min−1 ramp to 240°C, and then held at the temperature for about 15 min. The injector and detector temperature was 250°C, the carrier gas was He (1 ml min−1, split ratio 1:50), and the samples and n-alkanes (consecutive C8-C40, purchased from AccuStandard, ( www.accustandard.com ) were diluted in acetone (injection of 2 µL). Mass spectra were recorded at 70 eV, and the mass range was m/z 30–600 amu ( Dalila et al. 2012 ). The compounds were identified by comparing their retention indices (Kovats indices) (Santos et al. 2011) with those of known compounds and their mass spectra with those stored in the MS database (NIST98 MS DATA). The relative percentage amounts were obtained directly from the GC peak areas.

### Fumigant activity

The fumigant bioactivities of EOs were detected with the sealing jar method ( Deng et al. 2004 ). Whatman No.1 filter papers were cut in filter paper strips (1.5 cm × 5 cm) and suspended in a 250 mL jar after adding 3.5 µL EO or acetone (control) on each strip (concentration of 14 µL/L). Thirty adults were added, and the vial quickly was covered with sealing plastic film. Each treatment was set for triplications and placed at 27 ± 2°C and 70 ± 5% RH. Mortalities and corrected mortalities after 48 hr and 96 hr were corrected for control mortality by using Abbott’s (1925) formula. EOs with stronger activities were screened out and their toxicities were assayed on 6th instars and adults at the concentrations of 2, 4, 8, 16, 32, and 0 (control) µL/L. The LC50 and LC95 (lethal concentration) values and their 95% confidence intervals (CI) values 48 hr and 96 hr after treatment were calculated with POLO 2.0 (LeOra Software, ( www.leorasoftware.com ) respectively.

### Contact activity and toxicity

The contact activities of EOs on 6th instar *A. diaperinus* were assayed with an impregnated paper assay ( Stefanazzi et al. 2011 ). EOs were diluted with acetone and applied to Whatman No. 1 filter paper (6 cm diameter) at a 40 µg/cm2 concentration, using 1 mL acetone as the control. When the solution had absolutely volatilized about 15 min, 30 6th instar *A. diaperinus* were placed into a glass Petri dish with teflon coated on the inner wall to prevent escaping. Mortality and corrected mortality were calculated 24 hr and 48 hr posttreatment. Contact toxicities of EOs were assayed at the concentrations of 5 µg/cm2, 10 µg/cm2, 20 µg/cm2, 40 µg/cm2, 80 µg/cm2, 160 µg/cm2 and 0 (control), and all insect were cultivated at 27 ± 2°C and 70 ± 5% RH. All treatments were set for triplications. The LC50 and LC95 values and their 95% CI after 24 hr and 48 hr were calculated as the description of fumigant activity.

### Repellent activity

The repellent activities of EOs on 6th instar *A. diaperinus* were determined as described by Wang et al (2006) and Yao et al (2008) with some modifications. Whatman No. 1 filter paper (diameter 12.5 cm) was cut in half, EOs were applied to half of a filter-paper disc as uniformly as possible, and other half of the filter paper was treated with acetone alone as the control. Three concentrations, 200, 400, 800 µg/cm2, were held by dissolving different volumes of EO in L ml acetone. The treated and control half discs were air-dried a tabout 20°C for 15 min to evaporate the solvent completely. Treated and untreated halves were attached to their opposites using adhesive tape and fixed in Petri dishes (diameter 12.5 cm) with Teflon coated on the inner wall to prevent escaping. Thirty 6th instar larvae were released at the center of each filter paper disc. The dishes were then covered and held in an incubator at room temperature, and three replications were set for each concentration. After 12 , 24, and 48 hr, the number of larvae present at each amount of treated or control halves were calculated, and the distribution coefficient was calculated as follows:

$\text{DC} = \frac{C - T}{C + T} \times 100\%$

where *C* is the number of insects present on the control areas of the discs and *T* is the number of insects present on the treated side.

### AChE Activity Assay

The effects of EOs on AChE activities of 6th instar *A. diaperinus* were assayed with the colorimetric method as described by Abdelgaleil et al (2009) and Yeom et al (2012) with some modifications. Fifteen 6th instars larvae treated with *C. limonum* , *L. cubeba* , and *A. sativum* EOs or nothing (control) were quick-frozen with liquid nitrogen at 6, 12, 24, 36, 48, 60, 72, and 96 hr after treatment and separately homogenized in 10 mL of 0.1 M icecold phosphate buffer (pH 7.0) using a mortar. Homogenates were centrifuged (7,000 rpm for 20 min at 0ºC), and supernatants were used as the enzyme source for determination of AChE activity, using acetylcholine bromide as substrate. Enzyme aliquots (50 µL) and DTNB (100 µL of 0.01 M) were added to 0.1 M phosphate buffer (pH 8.0, 2.8 mL). Mixtures were incubated at 37ºC for 15 min. Reactions were started by adding acetylcholine bromide (30 µL) followed by incubation at 37ºC for 10 min. Absorbance was measured at 412 nm using a UV 2000-Spectrophotometer (NanoDrop, ( www.nanodrop.com ). Timecourses of AChE activity were examined, and each treatment was corrected by blanks for nonenzymic hydrolysis. All the experiments were performed in triplicate. Inhibition percentage of AChE activity was calculated as follows:

$\text{AChE} \,\text{inhibition} (\%) = \frac{OD_{B} - OD_{T}}{OD_{B}} \times 100$

where ODB is the optical density of blank enzyme and ODT is the optical density of treatment.

### Statistical analyses

In the experiment, the fumigant, contact, and repellent activities and inhibition on AChE activity of EOs were compared using analysis of variance (ANOVA) followed by Duncan’s test for multiple -comparison ( *P* < 0.05). The recorded mortality data in fumigant and contact toxicity tests were adjusted for mortality in the control using Abbott’s formula, analyzed by oneway ANOVA, and means were compared using Duncan’s test at *P* < 0.05 through an SPSS version 17.0 software package (IBM, ( www.ibm.com ) in Microsoft Windows 7 operating system (( www.microsoft.com ). The figure of inhibition on AChE activity was drawn by SigmaPlot version 10.0 software (( www.sigmaplot.com ).

## Results

### The main ingredients of test EOs

According to the GC-MS data ( Table 1 ), the main ingredient of *C. limonum* was limonene, and *L. cubea* was mainly constituted of D-limonene, (Z)-3, 7-dimethyl-, 2, 6-octadienal, and (E)-3, 7-dimethyl-, 2, 6-octadienal. *C. cassia* mainly contained methyl salicylate and (E)-cinnamaldehyde, and *A. sativum* mostly was composed of diallyl disulphide, di-2-propenyl trisulfide, and di-2-propenyl tetrasulfide.

**Table 1.**
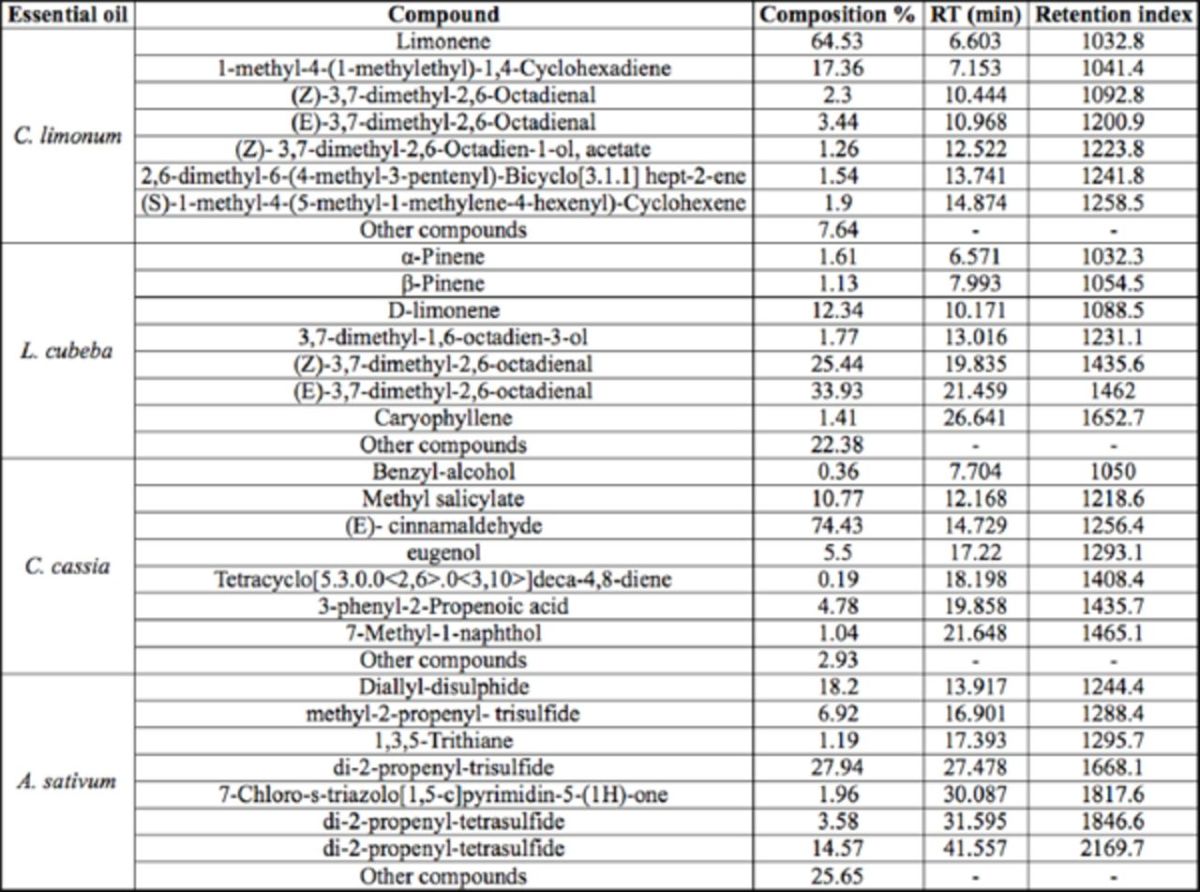
Chemical constituent of essential oils extracted from *C. limonum, L. cubeba, C. cassia* , and *A. sativum.* RT = retention time.

### Fumigant activity

Fumigant activities of EOs on the adults of *A. diaperinus* showed that *A. sativum* had the strongest fumigant activities 48 to 96 hr posttreatment, followed by *C. limonum* , *C. cassia* , and *L. cubeba* ( Table 2 ). No insect mortality was observed in the control.

**Table 2.**
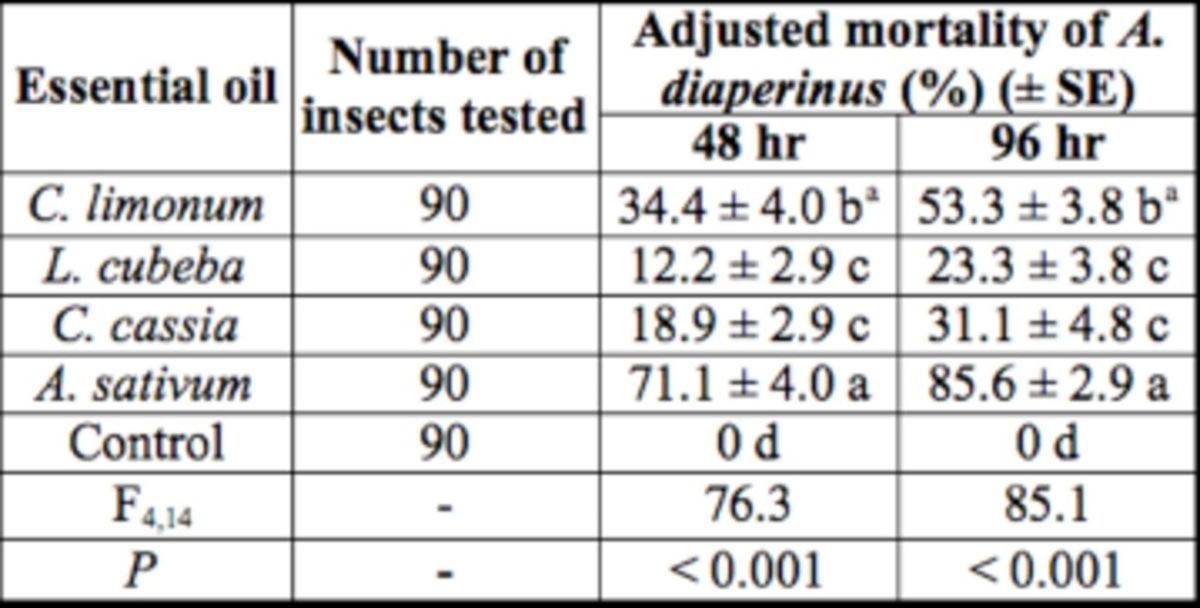
The fumigant activity of EOs on *A. diaperinus* adults.

aMeans within a column followed by the same letters are significantly different ( *P <* 0.05) as determined by Duncan’s test.

The toxicities of *A. sativum* and *C. limonum* seemed to be stronger on 6th instars larvae ( Table 3 ) than on adults ( Table 4 ). Nevertheless, the toxicities of the EOs were almost equitoxic (overlapping confidence intervals) at 96 hr after treatment. No insect mortality was observed in the control.

**Table 3.**
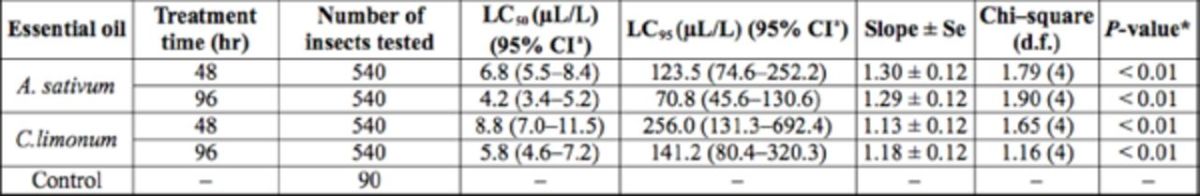
LC50 values from fumigant activity of *A. sativum* and *C. limonum* to 6th instar *A. diaperinus.*

aLC50 or LC95 values are considered significantly different when the 95% confidence intervals (CI) do not overlap. *Goodness-of-fit test is significant at *P* < 0.05.

**Table 4.**
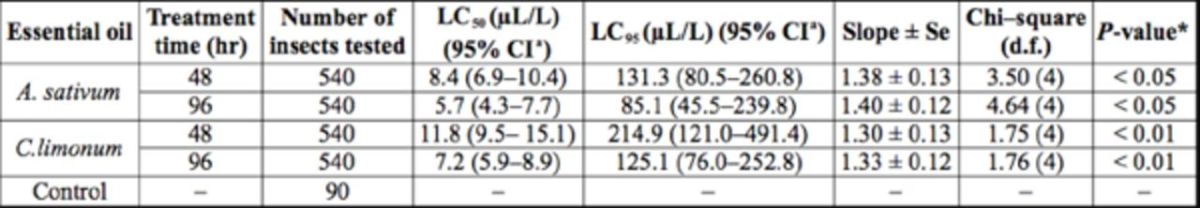
LC50 values from fumigant activity of *A. sativum* and *C. limonum* to *A. diaperinus* adults.

aLC50 or LC95 values are considered significantly different when the 95% confidence intervals (CI) do not overlap. *Goodness-of-fit test is significant at *P* < 0.05.

### Contact activity

The contact activities of EOs on 6th instar *A. diaperinus* are shown in Table 5 . The activity of *A. sativum* was the highest, followed by *L. cubeba* .. However, the contact activities of the other two EOs were not ideal, causing < 50% adjusted mortality during the experiment period.

**Table 5.**
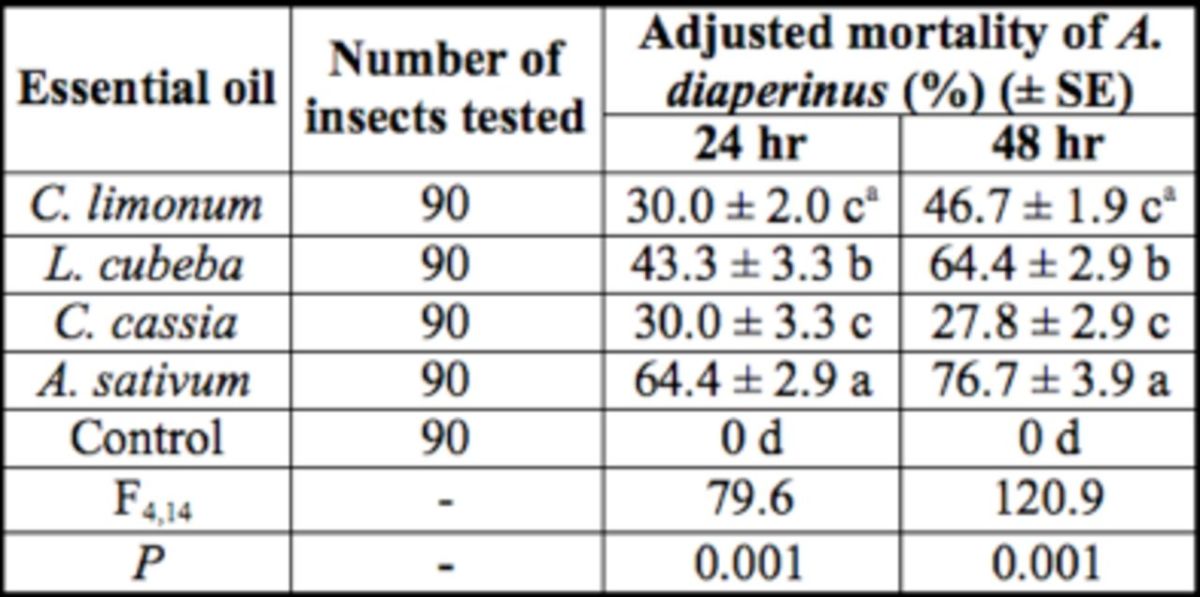
The contact activity of EOs on 6th instar *A. diaperinus* .

aMeans within a column followed by the same letters are not significantly different ( *P >* 0.05) as determined by Duncan’s test.

The contact toxicity of *A. sativum* on 6th instars of *A. diaperinus* was stronger than that of *L. cubeba* . The lower LC50 value of *A. sativum* was recorded 24 hr after treatment and was not equitoxic with that of *L. cubeba* (not overlapping confidence intervals). Nevertheless, the toxicity of *L. cubeba* was enhanced quickly and seemed to be equitoxic with that of *A. sativum* (48 hr after treatment (overlapping confidence intervals) ( Table 6 ). No insect mortality was observed in the control.

**Table 6.**
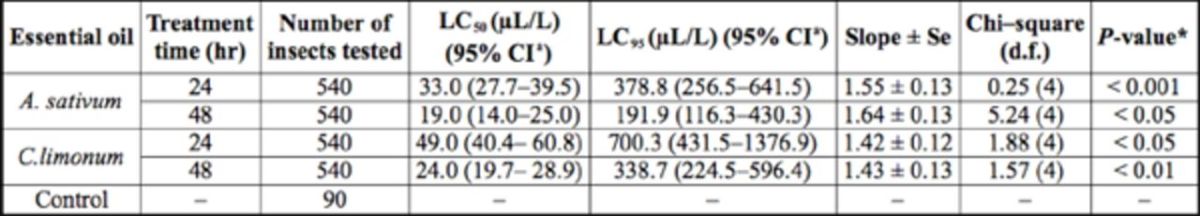
The contact toxicity of EOs on 6th instar *A. diaperinus* .

aLC50 or LC95 values are considered significantly different when the 95% confidence intervals (CI) do not overlap. *Goodness-of-fit test is significant at *p* < 0.05.

### Repellent activity

The repellent activity of *A. sativum* was the strongest among the tested oils, followed by *L. cubeba* (both significant). The repellent activities of the other two EOs were not significant 12, 24, and 48 hr after treatment ( Table 7 ). Nevertheless, the repellent activities of all EOs decreased with the elongation of experiment.

**Table 7.**
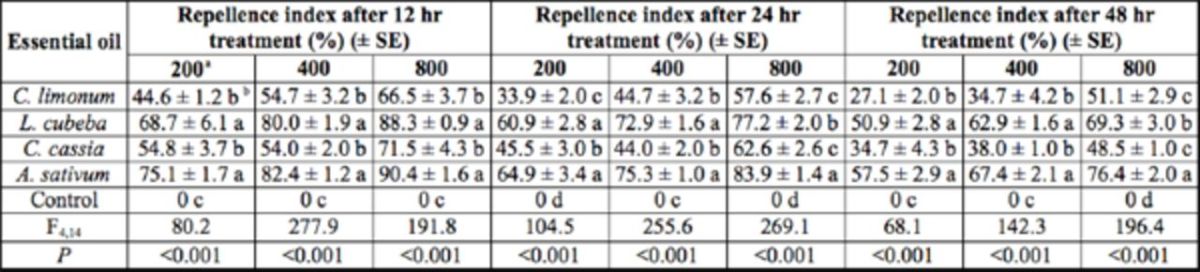
Repellent activity of EOs on 6th instar *A. diaperinus* .

aµg/cm2 filter paper. Means in the same column followed by the same letters differ significantly ( *P <* 0.05) according to ANOVA. The number of insects tested for each treatment was 90.

### Inhibiton of EOs on AChE activity

**Figure 1.**
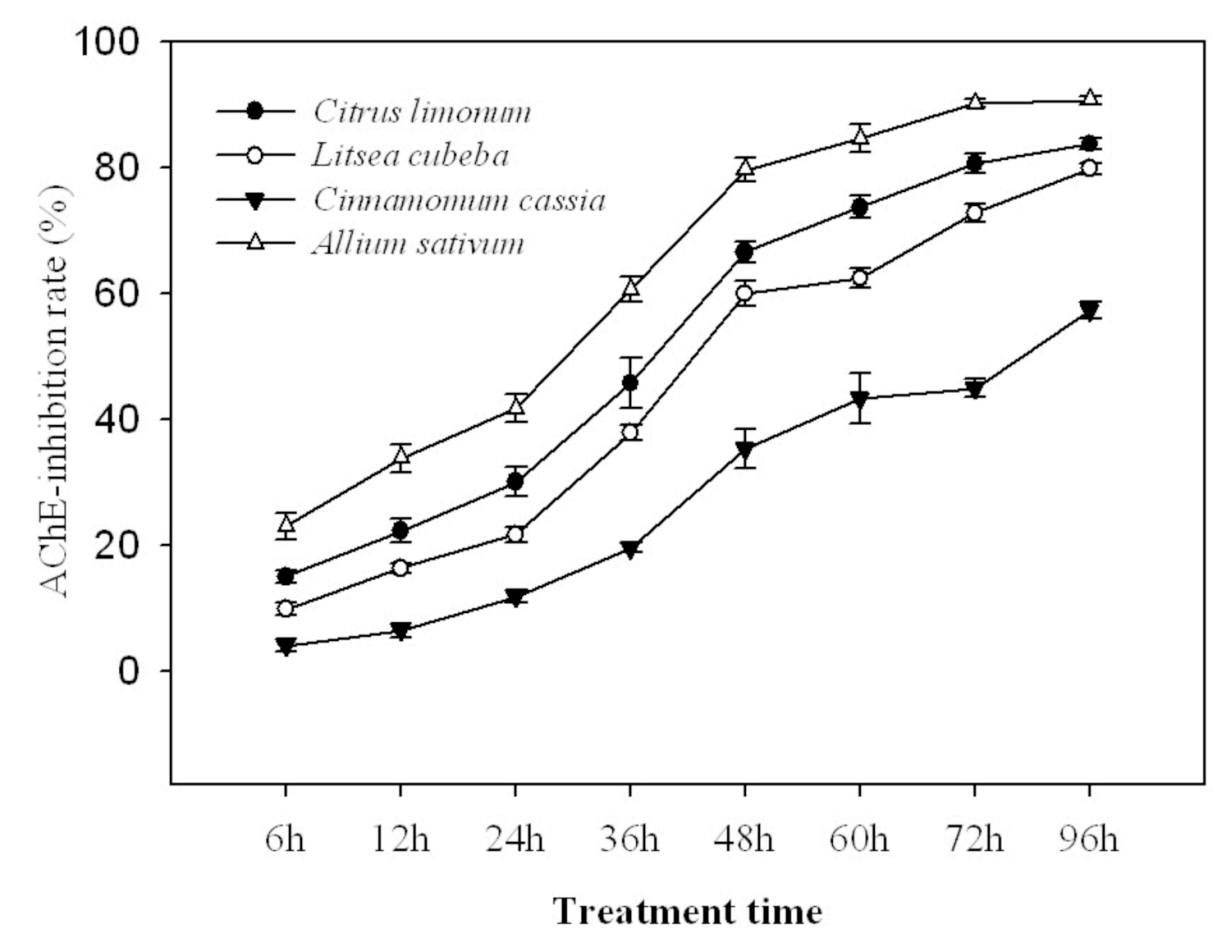
The timecourse of EOs on AChE activity of 6th instar *A. diaperinus* . Note: The AChE activity of tested EOs on 6th instar *A. diaperinus* was the average values of triplication. High quality figures are available online.

*A. sativum* showed the strongest inhibition on AChE activity in 6th instar *A. diaperinus* , followed by *C. limonum* , *L. cubeba* , *C. cassia.* All the essential oilss inhibition activities were significant ( *P* < 0.01). Meanwhile, the inhibitions of EOs on AChE activity also became stronger and stronger with time and displayed timecourse effects. Inhibitions of all oils except for *A. sativum* were < 50% at 36 hr, but quickly enhanced from 48 hr to 96 hr ( Figure 1 ).

## Discussion

The results on the compositions of the *A. sativum* ( Park and Shin 2005 ; Almeida et al. 2009 ; Yang et al. 2010 ; Martinez-Velazquez et al. 2011 ), *C. limonum* ( Almansa et al. 2002 ; Ponce et al. 2004 ; Moreira et al. 2005 ; Pouvova et al. 2008 ; Rekha and Vijayalakshmi 2010 ), and *L. cubeba* ( Jiang et al. 2009 ; Seo et al. 2009 ) essential oils are in agreement with literature data on other insects. The fumigant, contact, and repellent activities of the tested EOs on adults and/or nymphal stages of *A. diaperinus* in present study showed that essential oils obtained from Sichuan Province acted against *A. diaperinus* . Furthermore, the efficacies of fumigant and contact activities were relatively enhanced with increasing amount of doses and exposure times. Similar findings were also shown in other investigations showing the toxic efficacy of EOs from some other aromatic plant against insects and mites ( Benzi et al. 2009 ).

Some ingredients (such as sulphide, limonene, pinene, etc.) from EOs have been shown to act as the major role of insecticidal activities, including fumigant, contact, and repellent. Yang et al. (2010) reported that *A. sativum* used against adult rice weevils, *Sitophilus oryzae* (L.), and red flour beetles, *Tribolium castaneum* (Herbst), could reduce the survival of eggs or larvae to adult stage. Gusmão et al. (2013) reported the main ingredients of EOs extracted from *Eucalyptus staigeriana* were limonene (28.75%), geranial (15.20%), and neral (12.16%), and from *Foeniculum vulgare* were limonene (41.82%), (E)-anethole (17.91%), and α-pinene (11.13%), and contact and fumigation toxicities of *F. vulgare* and *E. staigeriana* on the adults of *Callosobruchus maculates* had LC50 values of 178.13, 345.57 µg/mL and 2.58, 7.85 µL/L of air, respectively. Yeom et al. (2013) also researched 11 kinds of Myrtaceae plant essential oils, including *Eucalyptus polybractea* , *Eucalyptus smithii* , and so on, and they displayed 100% fumigant toxicity against adult male German cockroaches (7.5 mg/L air). The essential oil constituents, including terpinolene, α-terpinene, and terpinen-4-ol, demonstrated strong fumigant toxicity against adult males and females, and eugenol, isoeugenol, methyl eugenol, and terpinen-4-ol showed strong contact toxicity against adult males of *B. germanica.* According to our results, the compounds of sulphide (diallyl-disulphide, methyl-2-propenyl-risulfide, di-2-propenyltetrasulfide, etc.), limonene (limonene, D-limonene), and pinene ( *α* -Pinene, *β* -Pinene) were the main ingredients of EOs extracted from *A. sativum* , *C. limonum* , and *L. cubeba* and could lead to the insecticidal activities on tested pests.

AChE is an important target for insecticides, and inhibition by phytochemicals from plant essential oils has been reported in previous research ( Abdelgaleil et al. 2009 ; Kang et al. 2013 ). Yeom et al. (2013) found isoeugenol from Myrtaceae plant essential oils exhibited inhibition activity against male acetylcholinesterase, with IC50 values of 0.22 mg/mL. Kim et al. (2013) also revealed some compounds from Apiaceae essential oils on acetylcholinesterase inhibitory activity of *Sitophilus oryzae* and found that *α* -Pinene showed the highest inhibition rate (97.36%), followed by *β* -pinene (54.96%) and limonene (51.23%) at a concentration of 1 mg/mL. The IC50 of α-pinene reached 0.019 mg/mL. Based on our data, the EOs of *A. sativum* , *C. limonum* , and *L. cubeba* all displayed good inhibition on AChE activity of 6th instar *A. diaperinus* . We did not detect the inhibition on AChE activity and IC50 of singular ingredients on the tested pest, which could lead to insecticidal activity (including sulphide, limonene and pinene, eugenol), but we can assume how these compounds function based on the previously mentioned reports.

In general, our results indicate that EOs of *A. sativum* , *C. limonum* , and *L.cubeba* and their components could be developed for managements of *A. diaperinus* . For the practical use of these oils and their constituents as new control agents, the safety of the oils and their components to humans and nontarget organisms and their mode of action should be further investigated.
